# Prediction of Thromboembolic Events in Heart Failure Patients in Sinus Rhythm: The Hong Kong Heart Failure Registry

**DOI:** 10.1371/journal.pone.0169095

**Published:** 2016-12-30

**Authors:** Jo-Jo Hai, Pak-Hei Chan, Yap-Hang Chan, Carol-Ho-Yi Fong, Duo Huang, Wen-Hua Li, Li-Xue Yin, Chu-Pak Lau, Hung-Fat Tse, Chung-Wah Siu

**Affiliations:** 1 Cardiology Division, Department of Medicine, The University of Hong Kong, Hong Kong SAR, China; 2 Endocrinology & Metabolism Division, Department of Medicine, The University of Hong Kong, Hong Kong SAR, China; 3 Department of Echocardiography & Non-invasive Cardiology Laboratory, Sichuan Academy of Medical Sciences & Sichuan Provincial People's Hospital, Chengdu, China; University of Miami School of Medicine, UNITED STATES

## Abstract

**Aim:**

Heart failure (HF) increases the risk of thromboembolic events (TE). Study in a Caucasian population has shown that the CHA_2_DS_2_-VASc score predicts TE among HF patients without atrial fibrillation. We sought to assess the usefulness of the CHA_2_DS_2_-VASc score in predicting TE in an Asian population and refine the scoring system to improve its predictability of TE among HF patients in sinus rhythm.

**Methods:**

A total of 1,202 consecutive patients who were admitted to our institution for new-onset HF from 2005 to 2012 and without atrial fibrillation or anticoagulation were retrospectively reviewed.

**Results:**

The mean age was 77.6 ± 12.2 years and 51.7% were female. After 36.2 ± 30.1 months, 113 (9.4%) developed TE. The annual incidence was 0.54%, 1.54%, 2.98% and 5.04% per year in those who had a CHA_2_DS_2_-VASc score of 1, 2–3, 4–5 and ≥6, respectively. In multivariate analysis, age ≥75 years [Hazard ratio (HR) 2.59, 95% confidence interval (CI) 1.23–5.46, *p* = 0.012), chronic ischemic heart disease (HR 1.54, 95% CI 1.02–2.31, *p* = 0.040) and chronic kidney disease (HR 1.66, 95% CI 1.09–2.53, *p* = 0.018) independently predicted TE. Incorporation of chronic ischemic heart disease and chronic kidney disease into the CHA_2_DS_2_-VASc score significantly increased the area under the Receiver Operating Curve from 0.57 (95% CI 0.54–0.59) to 0.61 (95% CI 0.55–0.66; *p* = 0.022).

**Conclusion:**

The CHA_2_DS_2_-VASc score is useful for stratification of the risk of TE among HF patients in sinus rhythm. Incorporation of chronic ischemic heart disease and chronic kidney disease into the score modestly improves its predictive value.

## Introduction

Heart failure (HF) is an emerging epidemic that affects 26 million people worldwide.[1] Although the condition is well-known for its poor prognosis due to pump failure and/or sudden death, significant morbidity and mortality also results from an increased risk of thromboembolism.[2–5] In fact, HF is the second most common cause of cardioembolic stroke after atrial fibrillation (AF).[6] Left ventricular dysfunction is associated with intra-cardiac stasis, endocardial and endothelial dysfunction, and a hypercoagulable state, all of which promote thrombus formation and subsequent embolization.[2, 7–9] In stark contrast to AF, in which long-term anticoagulation is shown to substantially reduce the risk of thromboembolic events (TE), randomized controlled trials in HF patients in sinus rhythm have failed to demonstrate a net clinical benefit of oral anticoagulation over antiplatelet agents or placebo.[10–13] In the largest Warfarin versus Aspirin in Reduced Cardiac Ejection Fraction Trial (WARCEF) that involved 2,305 HF patients in sinus rhythm, warfarin conferred a reduction in ischemic stroke by 48% compared with aspirin that was offset by an increase in major hemorrhage.[13] Nonetheless this may also suggest that there exists a high-risk subset of HF patients in sinus rhythm who may derive a net clinical benefit from oral anticoagulation therapy.

The CHA_2_DS_2_-VASc score is a risk stratification tool to predict TE among patients with non-valvular AF.[14–18] This simple clinical prediction rule has been well validated in different populations and is recommended by current guidelines for the stratification of patients with AF for antithrombotic therapy.[15–20] Recently, the CHA_2_DS_2_-VASc score has also been shown in a Danish registry to predict TE among HF patients in sinus rhythm.[4, 21] Nevertheless this has not been evaluated in other populations. Furthermore, since the CHA_2_DS_2_-VASc score is based on studies in AF populations[14], clinical parameters not included in the CHA_2_DS_2_-VASc score may have incremental value for the prediction of TE among HF patients in sinus rhythm. We therefore performed this study to 1) determine independent clinical predictors of TE among HF patients in sinus rhythm; 2) assess the usefulness of the CHA_2_DS_2_-VASc score in predicting TE in Asian HF patients; 3) assess the value of incorporating independent clinical predictors into the CHA_2_DS_2_-VASc score to predict TE in HF patients in sinus rhythm.

## Materials and Methods

### Study design

This was a retrospective observational study based on the Hong Kong Heart Failure Registry. The study protocol was approved by the local Institutional Review Board. Details of the registry have been described in a previous study.[22] In summary, patients at Queen Mary Hospital, Hong Kong who were diagnosed with new-onset HF based on the Framingham Heart Study criteria from January 2005 to April 2012, were identified via the computerized clinical management system.[23] Demographic data, cardiovascular risk factors, clinical presentation, echocardiographic findings and laboratory test results on admission were recorded and clinical outcomes were followed. Patients who were younger than 18 years of age, had incomplete follow-up data, or were prescribed anticoagulation were excluded. Prior myocardial infarction was defined as a myocardial infarction that occurred during or prior to the index hospitalization. Chronic ischemic heart disease was defined as either a significant coronary artery stenosis diagnosed by angiography or myocardial ischemia diagnosed by stress testing in those without prior myocardial infarction. Chronic kidney disease was defined as an estimated glomular filtration rate (eGFR) <60 ml/min/1.73m^2^ by the Modification of Diet in Renal Disease formula.[24] HF with preserved ejection fraction (HFPEF) was defined as HF with left ventricular ejection fraction (LVEF) ≥40%.[25] The CHA_2_DS_2_-VASc score of each patient at diagnosis of HF was calculated (C: congestive heart failure [1 point]; H: hypertension [1 point]; A_2_: age 65–74 years [1 point] and age ≥75 years [2 points]; D: diabetes mellitus [1 point]; S: prior stroke or transient ischemic attack [2 points]; V: vascular disease, defined as prior myocardial infarction or peripheral vascular disease [1 point]; and Sc: sex category = female [1 point]).[14]. The primary outcome was TE and included ischemic stroke, transient ischemic attack and peripheral thromboembolism. All diagnoses were adjudicated by two cardiologists in accordance with the updated consensus statements and guidelines.[24, 26–30]

### Statistical analysis

Continuous variables are expressed as mean ± standard deviation, and categorical variables are presented in frequency tables. Statistical comparison of continuous variables was performed using Student’s *t* test, and catagorical variables with Fisher’s exact test or Chi-square test. Kaplan-Meier survival analysis with the log-rank test was used to compare TE-free survival of different patient groups. Hazard ratio (HR) and 95% confidence interval (CI) of clinical variables to predict primary outcome among HF patients in sinus rhythm were determined by a multivariate Cox regression model using a p value <0.1 for inclusion. The prognostic performance of prediction models of TE was assessed using c-statistics and compared using the DeLong test. Internal validation of the final prediction model was evaluated by bootstrapping 1,000 random samples. The optimism was estimated by comparing the final model performance on each bootstrapped sample to that of the original data. The corrected area under the Receiver-Operating Characteristics (ROC) curve was computed by subtracting the optimism from the original area under the ROC curve. A 2-tailed *p* value <0.05 was considered statistically significant. Calculations were performed using SPSS software (version 21.0) and R package (version 3.3.1).

## Results and Discussion

**Fig 1:**
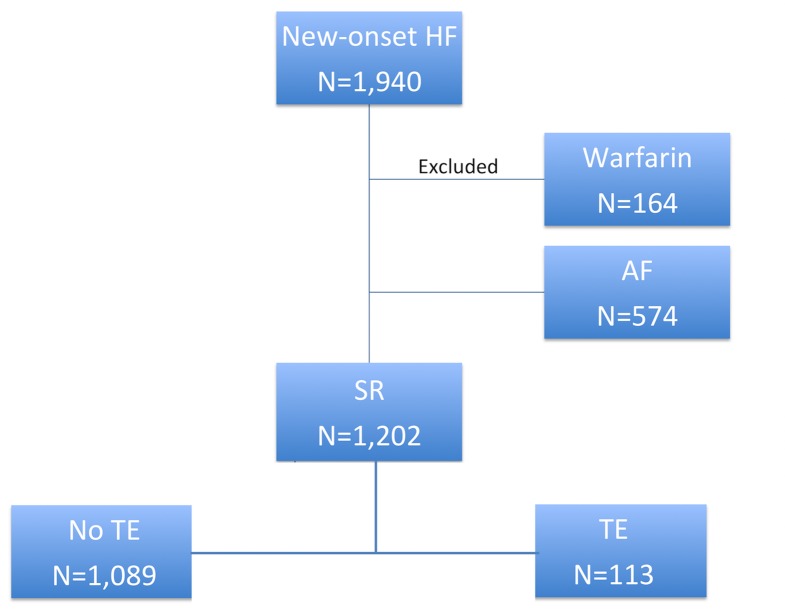
A flow chart showing selection, exclusion and clinical outcomes of our study population. SR–sinus rhythm.

Patient selection, exclusion and clinical outcomes are summarized in Fig 1. From January 2005 to April 2012, 1,940 patients were admitted to our hospital for new-onset HF. After excluding 164 patients (8.5%) who were prescribed warfarin, the final analysis included 1,776 patients. The mean age of the cohort was 78.7 ± 11.7 years and 965 (54.3%) were female. Of the 858 patients (48.3%) who had a technically adequate echocardiogram during the admission, the mean LVEF was 47.0 ± 16.0% and 59.3% of patients had a LVEF ≥40%, i.e., HFPEF.

### Clinical characteristics and outcomes of patients with and without AF

A total of 574 patients (32.3%) had prior or concomitant AF, and the remaining 1,202 were in sinus rhythm (67.7%). Table A in S1 File summarizes their clinical characteristics. After a mean follow-up of 36.2 ± 30.1 months, 190 patients with new-onset HF developed TE, of whom 169 had an ischemic stroke and 21 a transient ischemic attack. The annual incidence of TE in our cohort was 3.55% per year (95% CI: 3.41–3.69). Of the 190 TE, 77 developed in those with AF and 113 in patients in sinus rhythm. The annual incidence of TE among HF patients with AF and in sinus rhythm was 5.23% per year (95% CI 4.88–5.62) and 2.91% per year (95% CI 2.79–3.05), respectively. Fig A in S1 File depicts the Kaplan-Meier survival analysis comparing TE-free survival of HF patients with AF and those in sinus rhythm.

### Predictors of TE among HF patients in sinus rhythm

Among HF patients in sinus rhythm, those who developed TE were more likely to have hypertension (82.3% vs. 72.5%, *p* = 0.025), chronic ischemic heart disease (40.7% vs. 24.0%, *p*<0.001) and chronic kidney disease (69.0% vs. 55.7%, *p* = 0.024. Table 1). They also had a higher CHA_2_DS_2_-VASc score (4.89 ± 1.40 vs. 4.52 ± 1.54, *p* = 0.014) and were more likely to be prescribed aspirin (54.9% vs. 43.5%, *p* = 0.022. Table 1). In univariate analyses, increasing age (age 65–74 years: HR 2.41, 95% CI1.07–5.41, *p* = 0.033; age ≥75 years: HR 3.10, 95% CI 1.50–6.43, *p* = 0.002), hypertension (HR 1.88, 95% CI 1.16–3.06, *p* = 0.010), diabetes mellitus (HR 1.46, 95% CI 1.01–2.11, *p* = 0.045), chronic ischemic heart disease (HR 1.95, 95% CI 1.34–2.83) and chronic kidney disease (HR 2.15, 95% CI 1.44–3.21, *p*<0.001) were associated with an increased risk of TE (Table 2). In multivariate analysis, increasing age (age ≥75 years: HR 2.59, 95% CI 1.23–5.46, *p* = 0.012), chronic ischemic heart disease (HR 1.54, 95% CI 1.02–2.31, *p* = 0.040) and chronic kidney disease (HR 1.66, 95% CI 1.09–2.53, *p* = 0.018) remained independently associated with TE (Table 2). Importantly, the use of aspirin, clopidogrel, and anti-HF medications such as betablockers, renal-angiotensin-aldosterone inhibitors and mineralocorticoid receptor antagonists was not associated with a reduced risk of TE in HF patients in sinus rhythm (Table 2).

**Table 1:** Baseline characteristics of 1,202 heart failure patients in sinus rhythm with and without thromboembolic events.

	All	With TE	No TE	*p-*value
	(n = 1,202)	(n = 113)	(n = 1,089)	
Age, (years)	77.6±12.2	78.7±9.2	77.5±12.5	0.195
Female, n (%)	622 (51.7)	61 (54.0)	561 (51.5)	0.623
Smoker, n (%)	399 (33.2)	34 (30.1)	365 (33.5)	0.529
Drinker, n (%)	163 (13.6)	17 (15.0)	146 (13.4)	0.664
Hypertension, n (%)	883 (73.5)	93 (82.3)	790 (72.5)	0.025*
Diabetes mellitus, n (%)	473 (39.4)	53 (46.9)	420 (38.6)	0.086
Chronic ischemic heart disease, n (%)	308 (25.6)	46 (40.7)	262 (24.0)	<0.001*
Prior myocardial infarction, n (%)	78 (6.5)	9 (8.0)	69 (6.3)	0.545
Peripheral vascular disease, n (%)	45 (3.7)	8 (7.1)	37 (3.4)	0.064
Prior ischemic stroke / TIA, n (%)	160 (13.3)	16 (14.2)	144 (13.2)	0.771
Availability of echocardiography	583 (48.5)	61 (54.0)	522 (47.9)	0.236
LVEF^#^, (%)	45.9±16.4	47.9±14.1	45.7±16.6	0.252
HFPEF^#^, n (%)	254 (56.4)	36 (59.0)	293 (56.1)	0.685
eGFR, ml/min/1.73m^2^, (%)	57.1±30.4	51.8±24.7	57.6±30.9	0.022*
Chronic kidney disease, n (%)	685 (57.0)	78 (69.0)	607 (55.7)	0.024*
CHA_2_DS_2_-VASc score	4.56±1.53	4.89±1.40	4.52±1.54	0.014*
1	43 (3.6)	1 (2.3)	42 (97.7)	
2–3	229 (19.1)	15 (6.6)	214 (93.4)	
4–5	629 (52.3)	58 (9.2)	571 (90.8)	
≥6	301 (25.0)	39 (13.0)	262 (87.0)	
Medications, n (%)				
Aspirin	536 (44.6)	62 (54.9)	474 (43.5)	0.022*
Clopidogrel	61 (5.1)	7 (6.2)	54 (5.0)	0.503
Betablockers	480 (39.9)	53 (46.9)	427 (39.2)	0.130
ACEI/ARB	619 (68.9)	61 (54.0)	558 (51.2)	0.621
MRA	44 (3.7)	3 (2.7)	41 (3.8)	0.792
Frusemide	965 (80.3)	91 (68.4)	874 (80.3)	1.000
Insulin	107 (8.9)	10 (8.8)	97 (8.9)	1.000
Statin	336 (28.0)	26 (23.0)	310 (28.5)	0.270

*p<0.05.

^#^Calculation was based on 349 patients with AF and 603 patients without AF who had LVEF measured on admission.

TIA–Transient ischemic attack; ACEI–angiotensin-converting enzyme inhibitors; ARB–angiotensin receptor blockers; MRA–mineralocorticoid receptor antagonists.

**Table 2:** Univariate and multivariate predictors of thromboembolic events in 1,202 heart failure patients in sinus rhythm.

	Univariate analysis		Multivariate analysis	
	HR (95% CI)	*p-*value	HR (95% CI)	*p-*value
Age		0.008*		0.039*
<65	Reference		Reference	
65–74	2.41 (1.07–5.41)	0.033*	2.09 (0.92–4.71)	0.077
≥75	3.10 (1.50–6.43)	0.002*	2.59 (1.23–5.46)	0.012*
Female	1.05 (0.73–1.52)	0.795		
Smoker	0.86 (0.58–1.29)	0.476		
Drinker	1.05 (0.63–1.76)	0.845		
Hypertension	1.88 (1.16–3.06)	0.010*	1.43 (0.87–2.36)	0.157
Diabetes mellitus	1.46 (1.01–2.11)	0.045*	1.20 (0.81–1.78)	0.359
Chronic ischemic heart disease	1.95 (1.34–2.83)	0.001*	1.54 (1.02–2.31)	0.040*
Prior myocardial infarction	1.33 (0.67–2.63)	0.409		
Peripheral vascular disease	2.05 (1.00–4.21)	0.050	1.69 (0.81–3.53)	0.165
Prior ischemic stroke / TIA	1.41 (0.83–2.39)	0.205		
HFPEF^#^	0.94 (5.64–1.57)	0.810		
Chronic kidney disease	2.15 (1.44–3.21)	<0.001*	1.66 (1.09–2.53)	0.018*
Medications				
Aspirin	1.39 (0.96–2.02)	0.081	1.18 (0.80–1.76)	0.401
Clopidogrel	1.12 (0.52–2.41)	0.772		
Betablockers	1.17 (0.81–1.69)	0.404		
ACEI/ARB	0.85 (0.59–1.23)	0.390		
MRA	0.61 (0.19–1.92)	0.398		
Frusemide	0.89 (0.56–0.14)	0.634		
Insulin	1.18 (0.62–2.26)	0.616		
Statin	0.80 (0.52–1.24)	0.316		

*p<0.05.

^#^Calculation was based on 349 patients with AF and 603 patients without AF who had LVEF measured on admission.

TIA–transient ischemic attack; ACEI–angiotensin-converting enzyme inhibitors; ARB–angiotensin receptor blockers; MRA–mineralocorticoid receptor antagonists.

### The CHA_2_DS_2_-VASc score and TE among HF patients in sinus rhythm

**Fig 2:**
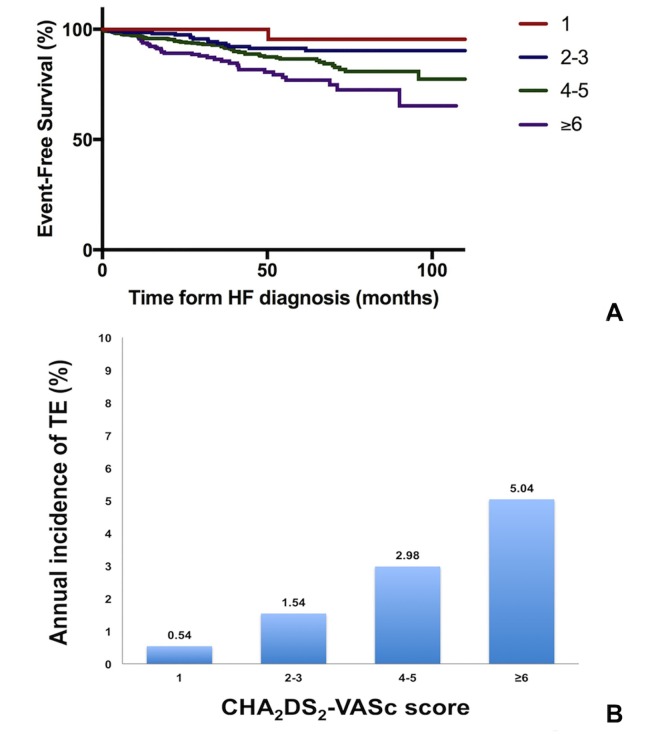
Risk of thromboembolic events among heart failure patients in sinus rhythm according to their CHA_2_DS_2_-VASc score. (A) Kaplan-Meier curves for thromboembolic event-free survival. Log-rank: 19.714. *P*<0.001. (B) Annual incidence of thromboembolic events. CHA_2_DS_2_-VASc = 1: 0.54% per year (95% CI 0.45–0.67); CHA_2_DS_2_-VASc = 2–3: 1.54% per year (95% CI 1.41–1.70); CHA_2_DS_2_-VASc = 4–5: 2.98% per year (95% CI 2.81–3.18); CHA_2_DS_2_-VASc ≥6: 5.04% per year (95% CI 4.59–5.60).

**Fig 3:**
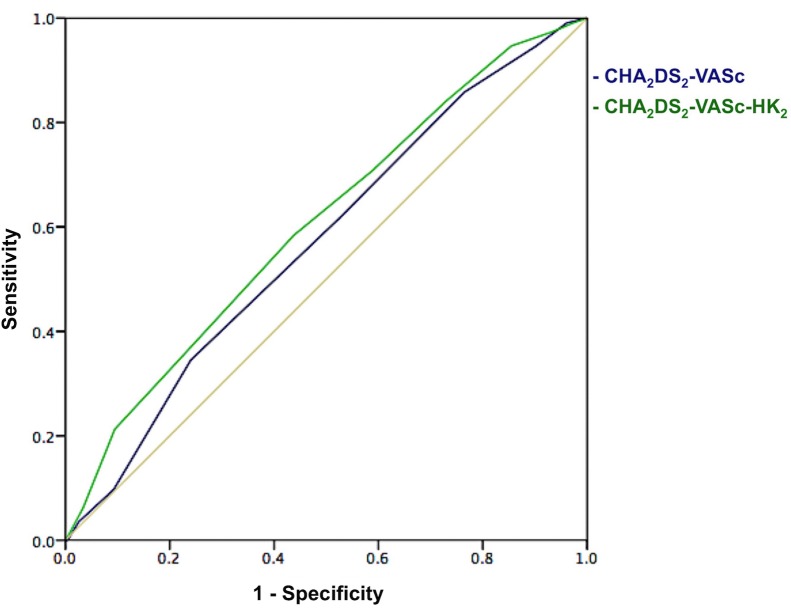
Receiver-Operating Characteristics curves for the CHA_2_DS_2_-VASc and the CHA_2_DS_2_-VASc-HK_2_ score to predict thromboembolic events among heart failure patients in sinus rhythm. The area under the curve for the CHA_2_DS_2_-VASc score was 0.57 (95% CI 0.54–0.59) and that for the CHA_2_DS_2_-VASc-HK_2_ score was 0.61 (95% CI 0.58–0.63). A significant improvement in the area under the curve was noticed after incorporation of chronic ischemic heart disease and chronic kidney disease into the CHA_2_DS_2_-VASc score (*p* = 0.022).

Kaplan-Meier survival analysis showed that the TE-free survival of HF patients in sinus rhythm reduced with increasing CHA_2_DS_2_-VASc score (log-rank *p*<0.001. Fig 2A). As shown in Fig 2B, the annual incidence of TE increased with an increasing CHA_2_DS_2_-VASc score. Specifically, for patients who had a CHA_2_DS_2_-VASc score of 1, i.e. no other risk factor other than HF alone, the annual incidence of TE was 0.54% per year (95% CI 0.45–0.67). For those who had a CHA_2_DS_2_-VASc score of 2–3, the annual incidence of TE was 1.54% per year (95% CI 1.41–1.70). Nevertheless for patients who had a CHA_2_DS_2_-VASc score of 4–5 and ≥6, the annual incidence of TE was as high as 2.98% (95% CI 2.81–3.18) and 5.04% (95% CI 4.59–5.60) per year, respectively. The area under the ROC curve for the CHA_2_DS_2_-VASc score to predict TE was 0.57 (95% CI 0.54–0.59. Fig 3).

### Modification of the CHA_2_DS_2_-VASc score to improve prediction of TE among HF patients in sinus rhythm

**Fig 4:**
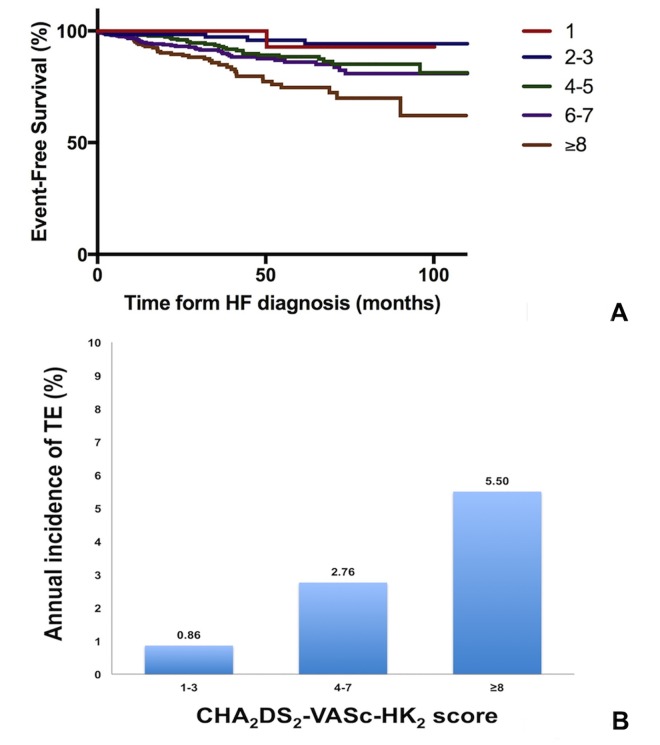
Risk of thromboembolic events among heart failure patients in sinus rhythm according to their CHA_2_DS_2_-VASc-HK_2_ score. (A) Kaplan-Meier curves for thromboembolic event-free survival. Log-rank: 25.896. *P*<0.001. (B) Annual incidence of thromboembolic events. CHA_2_DS_2_-VASc-HK_2_ = 1–3: 0.86% per year (95% CI 0.78–0.96); CHA_2_DS_2_-VASc-HK_2_ = 4–7: 2.76% per year (95% CI 2.61–2.92); CHA_2_DS_2_-VASc-HK_2_ ≥8: 5.50% per year (95% CI 4.99–6.12).

Based on the additional independent predictors of TE identified in the multivariate cox regression analysis, we developed the CHA_2_DS_2_-VASc-HK_2_ by incorporating 1) chronic ischemic heart disease, and 2) chronic kidney disease into CHA_2_DS_2_-VASc score as follows: C: congestive heart failure [1 point]; H: hypertension [1 point]; A_2_: age 65–74 years [1 point] and age ≥75 years [2 points]; D: diabetes mellitus [1 point]; S: prior stroke or transient ischemic attack [2 points]; V: peripheral vascular disease and aortic disease [1 point]; and Sc: sex category = female [1 point]; H: ischemic heart disease, including myocardial infarction or chronic ischemic heart disease [1 point]; K: chronic kidney disease [2 points]. In general, the TE-free survival of HF patients in sinus rhythm also reduced with an increasing CHA_2_DS_2_-VASc-HK_2_ score (log-rank *p*<0.001. Fig 4A). In particular, patients with a CHA_2_DS_2_-VASc-HK_2_ score of 1–3 had good TE-free survival, patients with a CHA_2_DS_2_-VASc-HK_2_ score of 4–7 had intermediate TE-free survival, and those with a CHA_2_DS_2_-VASc-HK_2_ score ≥8 had the worst TE-free survival. The annual incidence of TE was 0.86% (95% CI 0.78–0.96), 2.76% (95% CI 2.61–2.92) and 5.50% (95% CI 4.99–6.12) per year for patients who had a CHA_2_DS_2_-VASc-HK_2_ score of 1–3, 4–7 and ≥8, respectively (Fig 4B). The area under the ROC curve for the CHA_2_DS_2_-VASc-HK_2_ score to predict TE was superior to that of the CHA_2_DS_2_-VASc score [0.61 (95% CI 0.55–0.66) vs. 0.57 (95% CI 0.54–0.59), *p* = 0.022. Fig 3]. When assessed by Cox regression analysis, a CHA_2_DS_2_-VASc-HK_2_ score of 4–7 was associated with a 3-fold increase in risk of TE and a CHA_2_DS_2_-VASc-HK_2_ score ≥8 was associated with a 6-fold increase in the risk of TE among HF patients in sinus rhythm (Table 3).

**Table 3:** Prediction of thromboembolic events in 1,202 heart failure patients in sinus rhythm using the CHA2DS2-VASc score and the CHA2DS2-VASc-HK_2_ score.

	HR (95% CI)	*p-*value
CHA_2_DS_2_-VASc score^1^	1.27 (1.13–1.44)	<0.001*
CHA_2_DS_2_-VASc score^2^		0.001*
1	Reference	
2–3	2.9 (0.39–22.1)	0.300
4–5	5.4 (0.74–38.84)	0.095
≥6	9.0 (1.23–65.28)	0.030*
CHA_2_DS_2_-VASc-HK_2_ score^1^	1.28 (1.17–1.40)	<0.001*
CHA_2_DS_2_-VASc-HK_2_ score^2^		<0.001*
1–3	Reference	
4–7	3.14 (1.36–7.24)	0.007*
≥8	6.12 (2.58–14.49)	<0.001*

*p<0.05.

1. Continuous variable.

2. Categorical variable.

### Internal validation of the CHA_2_DS_2_-VASc-HK_2_ score

Internal validation of the final prediction model was performed as described. Cox regression analyses of 1000 bootstrapped samples resulted in the same independent predictors of TE (Table B in S1 File). The optimism-corrected area under the ROC curve was 0.61 (95% CI 0.55–0.66). We further compared the ability of the CHA_2_DS_2_-VASc-HK_2_ in predicting TE among patients with HFREF and HFPEF using *z*-test. The area under the ROC curve for the CHA_2_DS_2_-VASc-HK_2_ to predict TE in patients with HFREF and HFREF were not significantly different [0.63 (95% CI 0.52–0.73) and 0.70 (95% CI 0.60–0.80), respectively, (*p* = 0.368)].

## Discussion

In this study, we confirmed that the risk of TE increases with an increasing CHA_2_DS_2_-VASc score in Asian HF patients in sinus rhythm. We also established that chronic ischemic heart disease and chronic kidney disease are independent predictors of TE among HF patients in sinus rhythm, incorporation of which into the CHA_2_DS_2_-VASc score modestly improves its predictive value.

Observational studies and post-hoc analyses from HF trials have shown that HF patients in sinus rhythm are at high risk of TE.[2–5] In our study, although the risk of TE was lower among HF patients in sinus rhythm than those with AF, the annual incidence was 2.91% per year, markedly higher than the reported incidence of 1.45 per 1000 persons per year in the general population.[31] Nevertheless, the risk of TE is not the same among all HF patients in sinus rhythm. Prior studies of a Danish registry have shown that the risk of TE increases with an increasing CHA_2_DS_2_-VASc score.[4, 21] We found a similar difference in the annual incidence of TE among HF patients with different CHA_2_DS_2_-VASc score. More importantly, both our study and the Danish study showed that a CHA_2_DS_2_-VASc score of 1 was associated with only modestly increased risk of TE.[21] Even in those who had a CHA_2_DS_2_-VASc score of 2–3, the annual incidence of TE was still less than 2% per year. In these patients, the benefit of anticoagulation may not outweigh the risk of the therapy. This may partially explain the negative results from previous randomized controlled trials that tested the benefit of anticoagulation therapy among HF patients in sinus rhythm.[10–13] Future randomized study is needed to properly assess the value of anticoagulation therapy among the high-risk subgroup of HF patients.

In this study, chronic ischemic heart disease and chronic kidney disease were independent predictors of TE. By incorporating these parameters into the CHA_2_DS_2_-VASc score, we modestly increased its predictive value from 0.57 (95% CI 0.54–0.59) to 0.61 (95% CI 0.55–0.66; *p* = 0.022). Furthermore, the new CHA_2_DS_2_-VASc-HK_2_ score was able to stratify HF patients in sinus rhythm into low (annual incidence of TE <2% per year), intermediate (annual incidence of TE 2–5% per year) or high (annual incidence of TE >5% per year) thromboembolic risk. Nevertheless, the relatively low area under the ROC curve implies that there remain significant missing variables in the new scoring system. In a pooled analysis of two clinical trials largely consisting of patients with chronic HFREF, Abdul-Rahim and colleagues have shown that age, previous stroke, New York Heart Association class, diabetes mellitus treated with insulin and body mass index predicted stroke in HF patients without AF.[5] We did not include New York Heart Association class or body mass index in our analysis, as both parameters are dynamic and particularly inaccurate in patients with new-onset HF.[5] Furthermore, we did not find prescription of insulin predictive of TE, in contrast to the findings by Abdul-Rhaim et al.[5] This is not surprising, as prescription of insulin in real-life situation can be affected by many factors. Other than secondary oral drug failure and chronic renal failure, availability of new oral hypoglycemic agents, perceived risk of tight diabetic control, acceptability and practicability of insulin injection, all affect one’s decision on prescribing insulin to a patient.

More recently, Abdul-Rahim et al. have published another study comprising patients from two clinical trials of chronic HFPEF.[32] They have found that patients with HFREF and HFPEF share similar risk factors for stroke.[32] In addition, they have shown that the risk model derived from the HFREF cohort predicts stroke in patients with HFPEF with comparable *c*-index.[5, 32] Similarly, our study did not find any significant differences in the ability of the CHA_2_DS_2_-VASc-HK_2_ score in predicting TE among patients with HFREF and HFPEF. These findings suggest that although the risk factors for HFREF and HFPEF are different, the risk factors for TE in HF remain similar. Furthermore, post-hoc analyses of previous clinical trials have not consistently shown that LVEF is a risk factor for TE in patients with HF.[33–35] In addition, the study by Abdul-Rahim et al. has not shown that LVEF predicts TE in HF patients.[5] It is likely that other factors leading to the common pathophysiologic pathway of inflammation, hypercoagulability, endocardial and endothelial dysfunction play a more important role in TE among HF patients than intracardiac stasis associated with LV systolic dysfunction per se.[2, 7–9]

Although both the CHA_2_DS_2_-VASc score and the CHA_2_DS_2_-VASc-HK_2_ score demonstrated limited predictive ability of TE as shown in the ROC analyses, both scoring systems involve simple calculation by summing up objective clinical risk factors, which improves their applicability as a risk stratification tool. External validation of the CHA_2_DS_2_-VASc-HK_2_ score is required to assess the robustness of this scoring system in predicting TE among HF patients in sinus rhythm.

A major strength of this study is that complete records for all patients were available, such that all baseline and outcome variables were adjudicated. In addition, patients in our study were followed up for a considerably long period of time. However, our study also has limitations. First, not all patients in our study had an echocardiogram performed on admission. As a result, our study might be underpowered to evaluate the effect of LVEF on TE. Nevertheless, our finding that LVEF was not predictive of TE is echoed by the result of another study comprising patients of clinical trials.[5] Second, although the incidence of TE was much higher in the HF than the general population, the actual number of events in each of the HFREF and HFPEF group was small due to small sample size, which precluded detailed subgroup analyses. However, previous study by Abdul-Rahim et al. has shown that the risk factors for stroke in HFREF and HFPEF are similar. Furthermore, we did not find any significant differences in the ability of the CHA_2_DS_2_-VASc-HK_2_ score in predicting TE among patients with HFREF and HFPEF. Third, patients were not systemically followed up for the development of AF. It is possible that some patients had undiagnosed paroxysmal AF prior to the development of TE. Forth, it is usually not possible to delineate the actual mechanism of ischemic cerebrovascular events.[36] As a result, the exact proportion of cardioembolic stroke versus ruptured atherosclerotic plaque remains undetermined.

## Conclusions

HF, even without AF, is associated with a high incidence of TE. The CHA_2_DS_2_-VASc score is useful in stratifying thromboembolic risk among this group of patients. Incorporation of chronic ischemic heart disease and chronic kidney disease into the scoring system confers a modest but significant improvement in the ability to predict TE among HF patients in sinus rhythm.

## Supporting Information

S1 File **Table A. Baseline characteristics of heart failure patients with and without atrial fibrillation. Table B. Internal validation based on 1000 bootstrapped samples.** A) Multivariate predictors of thromboembolic events in 1,000 bootstrapped samples. B) Prediction of thromboembolic events in 1,000 bootstrapped samples using the CHA2DS2-VASc-HK_2_ score. **Fig A. Kaplan-Meier survival analysis of thromboembolic event-free survival among heart failure patients with and without atrial fibrillation.** Log-rank: 9.085. *P* = 0.003.
(PDF)

## Abbreviations

AF: atrial fibrillation
CI: confidence interval
eGFR: estimated glomerular filtration rate
HR: hazard ratio
HF: heart failure
HFPEF: heart failure with preserved ejection fraction
LVEF: left ventricular ejection fraction
ROC: Receiver-Operating Characteristics
TE: thromboembolic events
WARCEF: Warfarin versus Aspirin in Reduced Cardiac Ejection Fraction
