# Supplementary material for: Prediction of Thromboembolic Events in Heart Failure Patients in Sinus Rhythm: The Hong Kong Heart Failure Registry
Source: PLoS One. 2016 Dec 30;11(12):e0169095. doi: 10.1371/journal.pone.0169095 (PMC5201293; doi:10.1371/journal.pone.0169095)
Supplement: S1 File — Table A. Baseline characteristics of heart failure patients with and without atrial fibrillation. Table B. Internal validation based on 1000 bootstrapped samples. A) Multivariate predictors of thromboembolic events in 1,000 bootstrapped samples. B) Prediction of thromboembolic events in 1,000 bootstrapped samples using the CHA2DS2-VASc-HK2 score. Fig A. Kaplan-Meier survival analysis of thromboembolic event-free survival among heart failure patients with and without atrial fibrillation. Log-rank: 9.085. P = 0.003. (PDF) [file pone.0169095.s001.pdf]

Table A. Baseline characteristics of heart failure patients with and without atrial fibrillation.

	All (n=1,776)	No AF (n=1,202)	AF (n=574)	*p*-value
Age, (years)	78.7±11.7	77.6±12.2	81.0±10.2	<0.001
Female, n (%)	965 (54.3)	622 (51.7)	343 (59.8)	0.002*
Smoker, n (%)	543 (30.6)	399 (33.2)	144 (25.1)	0.001*
Drinker, n (%)	230 (13.0)	163 (13.6)	67 (11.7)	0.290
Hypertension, n (%)	1,269 (71.5)	883 (73.5)	386 (67.2)	0.007*
Diabetes mellitus, n (%)	647 (36.4)	473 (39.4)	174 (30.3)	<0.001*
Chronic ischemic heart disease, n (%)	470 (26.4)	308 (25.6)	162 (28.2)	0.250
Prior myocardial infarction, n (%)	101 (5.7)	78 (6.5)	23 (4.0)	0.037*
Peripheral vascular disease, n (%)	62 (3.5)	45 (3.7)	34 (5.9)	0.490
Prior ischemic stroke / TIA, n (%)	263 (14.8)	160 (13.3)	103 (17.9)	0.012*
Availability of echocardiography	858 (48.3)	583 (48.5)	275 (47.9)	0.839
LVEF[#], (%)	47.0±16.0	45.9±16.4	49.4±15.0	0.003*
HFPEF[#], n (%)	509 (59.3)	329 (56.4)	180 (65.5)	0.014*
eGFR, ml/min/1.73m^2, (%)	58.4±29.8	57.1±30.4	61.2±28.3	0.007*
Chronic kidney disease, n (%)	984 (55.4)	685 (57.0)	299 (52.1)	<0.001*
CHA_2DS_2-VASc score	4.60±1.51	4.56±1.53	4.71±1.47	0.045*
1	55 (3.1)	43 (3.6)	12 (2.1)	
2-3	325 (18.3)	229 (19.1)	96 (16.7)	
4-5	937 (52.8)	629 (52.3)	308 (53.7)	
≥6	459 (25.8)	301 (25.0)	158 (27.5)	
Medications, n (%)				
Aspirin	885 (49.8)	536 (44.6)	349 (60.8)	<0.001*
Clopidogrel	83 (4.7)	61 (5.1)	22 (3.8)	0.280
Betablockers	662 (37.3)	480 (39.9)	182 (31.7)	0.001*
ACEI/ARB	899 (50.6)	619 (68.9)	280 (48.8)	0.287
MRA	66 (3.7)	44 (3.7)	22 (3.8)	0.893
Frusemide	1,440 (81.1)	965 (80.3)	475 (82.8)	0.219
Insulin	133 (7.5)	107 (8.9)	26 (4.5)	0.001*
Statin	460 (25.9)	336 (28.0)	124 (21.6)	0.005*

*$p<0.05$.

[#]Calculation was based on 349 patients with AF and 603 patients without AF who had LVEF measured on admission.

TIA – transient ischemic attack; ACEI – angiotensin-converting enzyme inhibitors; ARB – angiotensin receptor blockers; MRA – mineralocorticoid receptor antagonists.

Table B. Internal validation based on 1000 bootstrapped samples.

**I) Multivariate predictors of thromboembolic events in 1,000 bootstrapped samples.**		
	**HR (95% CI)**	***p*-value**
Age		
<65	Reference	
65-74	2.09 (0.79-7.52)	0.074
≥75	2.59 (1.24-8.81)	0.014*
Hypertension, n (%)	1.43 (0.87-2.80)	0.160
Diabetes mellitus, n (%)	1.20 (0.78-1.84)	0.360
Chronic ischemic heart disease, n (%)	1.54 (0.999-2.41)	0.048*
Peripheral vascular disease, n (%)	1.69 (0.74-3.00)	0.118
Chronic kidney disease, n (%)	1.66 (1.07-2.69)	0.025*
Medications, n (%)		
Aspirin	1.18 (0.79-1.73)	0.417

**II) Prediction of thromboembolic events in 1,000 bootstrapped samples using the CHA2DS2-VASc-HK$_2$ score.**		
	**HR (95% CI)**	***p*-value**
CHA2DS2-VASc-HK2 score[1]	1.28 (1.17-1.41)	0.001*
CHA2DS2-VASc-HK2 score[2]		
1-3	Reference	
4-7	3.14 (1.41-12.21)	0.010*
≥8	6.12 (2.53-30.72)	0.001*

*p<0.05.

1. Continuous variable.

2. Categorical variable.

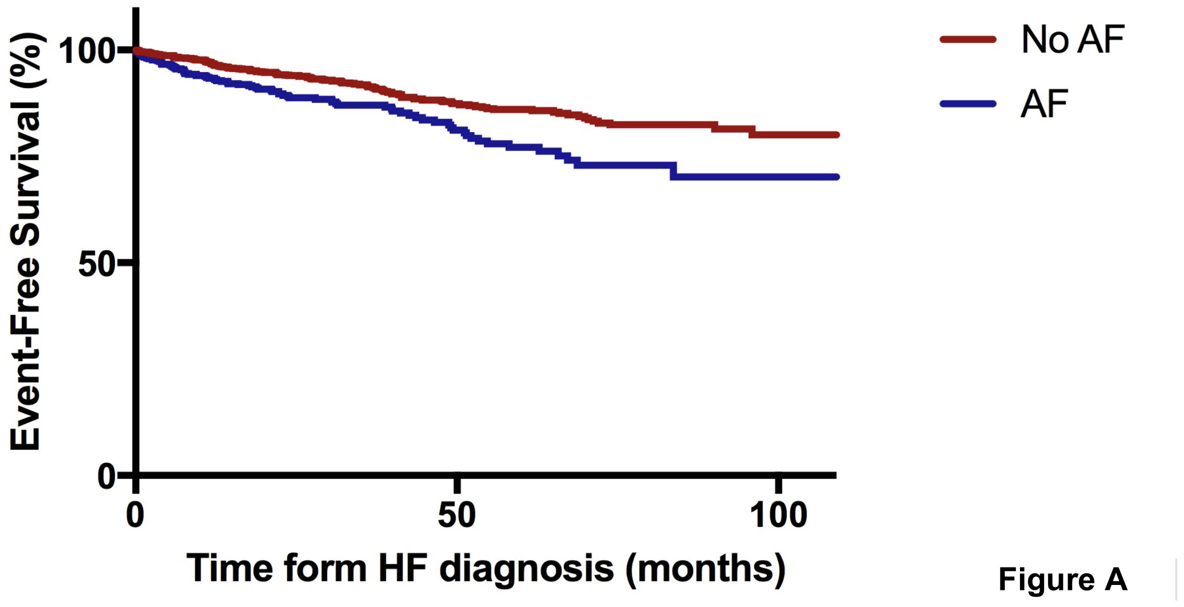

Figure A. Kaplan-Meier survival analysis of thromboembolic event-free survival among heart failure patients with and without atrial fibrillation. Log-rank: 9.085. *P*=0.003.
